# Joint Decision-Making and the Coordination of a Sustainable Supply Chain in the Context of Carbon Tax Regulation and Fairness Concerns

**DOI:** 10.3390/ijerph14121464

**Published:** 2017-11-27

**Authors:** Zhi Liu, Xiao-Xue Zheng, Ben-Gang Gong, Yun-Miao Gui

**Affiliations:** 1 College of Management Engineering, Anhui Polytechnic University, Wuhu 241000, China; liuzhi@nuaa.edu.cn (Z.L.); bggong@ahpu.edu.cn (B.-G.G.); ymgui789@163.com (Y.-M.G.); 2 Odette School of Business, University of Windsor, Windsor, ON N9B 3P4, Canada; 3 College of Transportation and Civil Engineering, Fujian Agriculture and Forestry University, Fuzhou 350002, China

**Keywords:** sustainable supply chain, carbon tax, fairness concern, sustainability level, low-carbon promotion

## Abstract

Carbon tax regulation and consumers’ low-carbon preference act as incentives for firms to abate emissions. Manufacturers can improve product sustainability and retailers can strengthen the promotion of low-carbon products as part of such abatement. Current incomplete rationality also affects product sustainability and low-carbon promotion level. In this context, we consider a supply chain with a manufacturer and a retailer and investigate the impacts of the manufacturer’s and the retailer’s fairness concerns on their production sustainability level, low-carbon promotion level and profitability. We also explore the coordination contract. The results show that the manufacturer’s and the retailer’s fairness concerns decrease their product sustainability and low-carbon promotion level, together with the profits of the system and the manufacturer. With regard to the retailer’s fairness concern, the product sustainability level and the manufacturer’s profit are lower; moreover, the low-carbon promotion level and the profits of the supply chain and the retailer are higher. A revenue-sharing contract can coordinate the supply chain perfectly; however, members’ fairness concerns increase the difficulty of coordination. Finally, the numerical results reveal that carbon tax regulation can encourage the manufacturer to enhance the product sustainability level. Further, the impacts on the low-carbon promotion level and firms’ profitability are related to the cost coefficients of product sustainability.

## 1. Introduction

With the degradation of the environment, public concern about environmental sustainability is growing worldwide [1]. Among these environmental problems, global warming is increasingly important. There is a developing consensus that carbon emissions (i.e., emissions of carbon dioxide and other greenhouse gases) are a main contributor to climate warming. To mitigate climate change, many countries have enacted legislation and designed mechanisms to curb carbon emissions, such as emission taxes, cap-and-trade policies, and deposit–refund schemes [2]. Of these mechanisms, carbon taxes are a means of price adjustment to guide the behavior of enterprises. In this regard, enterprises pay for each unit of their carbon emissions at a fixed tax rate [3]. Such carbon taxes have been applied in many countries. For example, British Columbia in Canada introduced a carbon tax in 2008. The tax is now CA$30/t CO_2_ and applied to almost all fossil fuel combustion in the province. Moreover, the tax has been verified as progressive [4]. Since 2012, Australia has imposed a carbon tax on industrial enterprises at a rate of AU$23/t CO_2_. As an environmental tax, a carbon tax imposes a charge on enterprises based on the “polluter pays” principle, which can create a financial incentive for enterprises to reduce their emission costs. The tax is proven to be effective for emission abatement, has few negative effects on economic growth, and is strongly advocated by experts and international organizations [5].

Under carbon tax regulation, enterprises can change their operations and invest in sustainability to decrease emissions [6]. The operations include production, inventory, and logistics. For example, after many suppliers reduced their packaging, Wal-Mart committed itself to cut 20 million metric tons of greenhouse gas emissions by 2015 [7]. Further, Hewlett-Packard (HP) reported that it had decreased toxic inventory releases into the air from 26.1 tons to 18.3 tons in 2010 [8]. Enterprises can also reduce their carbon emissions by investing in sustainability projects such as greener transportation fleets, energy-efficient warehousing, and environmentally friendly products (using greener materials and environmentally friendly manufacturing processes, a strategy referred to as product sustainability). For example, H&M has adopted new technologies to minimize carbon emissions and launched green-label products that the organization claims are produced in a sustainable way [9]. From the perspective of enterprises, improving product sustainability can not only decrease emission costs but also spur market demand. Moreover, because of consumers’ willingness to pay for more sustainable products at higher prices [10,11], enterprises may voluntarily invest in sustainability to abate emissions.

It is necessary for manufacturers and other affected parties (i.e., retailers) to make joint efforts to invest in sustainability. Improving product sustainability reduces the tax burden of manufacturers, which may result in lower wholesale prices and higher willingness-to-pay among consumers. Thus, retailers can also benefit from sustainability improvements. To this end, retailers have the motivation to offer manufacturers incentive schemes, such as revenue sharing, and to invest in low-carbon promotions of sustainable products. Retailers’ low-carbon promotions can also strengthen consumers’ awareness of the environmentally friendly features of products, which leads to a greater willingness to purchase sustainable products [12]. For example, Wal-Mat has established special zones in its stores that adopt an educational and promotional approach to low-carbon products and lifestyles, thereby enhancing consumers’ low-carbon product demands. Moreover, retailers’ low-carbon promotions can encourage manufacturers to launch incentive schemes for emission abatement. However, investing in sustainability and low-carbon promotion is always an arduous and costly endeavor for manufacturers and retailers; generally, the greater the product sustainability and low-carbon promotion, the higher the investment [13]. Thus, manufacturers and retailers must determine their optimal investment in emission abatement by comparing income with expenditure in terms of their efforts.

Besides the benefits and costs of investment in emission abatement, the fairness concerns of decision-makers can affect such investment. Research in behavioral economics has shown a significant incidence of cases in which enterprises are motivated by concerns of fairness in business relations [14]. Further, studies in economics in the past three decades have indicated that fairness plays an important role in developing channel relationships [15,16,17]. Thus, fairness concerns are a factor that decision-makers should not ignore when investing in emission abatement and the operational management of supply chains. Studies on the operational decisions of supply chains under carbon emission regulation have typically assumed that all channel members are perfectly rational, which means that such members care only about their monetary payoffs. This focus on monetary payoffs has produced some meaningful conclusions. For example, Yang et al. [7] show that revenue-sharing and cost-sharing mechanisms offered by a retailer can improve system efficiency and a manufacturer’s incentive for emission abatement under carbon tax regulation. Moreover, revenue sharing has an edge over cost sharing. Dong et al. [9] point out that the optimal profit of an entire supply chain is achieved at a wholesale price that is almost double that of the unit production cost. Further, the production quantity may unexpectedly increase the wholesale price under cap-and-trade regulation. It is unclear, however, whether these managerial prescriptions can be applied to a supply chain where some members care about monetary payoffs as well as fairness.

Based on the foregoing, this study investigates these problems of investment in emission abatement and operational decisions in the context of a manufacturer–retailer supply chain. The study also explores the coordination problem under carbon tax regulation, taking into account fairness concerns. Under carbon tax regulation, how do the fairness concerns of different members affect the incentives for emission abatement and the profits of all members in a supply chain? What has a greater influence on the supply chain: the retailer’s fairness concerns or those of the manufacturer? How does carbon tax regulation affect the incentives for emission abatement and the profits of all members in the supply chain? Is there a contract that can coordinate the supply chain? All these questions are relevant to this study. Further, the main contributions of the study are as follows. First, we investigate the abatement emission efforts of the manufacturer and retailer under carbon tax regulation, taking into account consumers’ low-carbon preference, the product sustainability level, and the low-carbon promotion level, all of which are used to describe the manufacturer’s and retailer’s efforts. Second, in terms of decision-makers’ bounder rationality, we study the impacts of the manufacturer’s and retailer’s fairness concerns on their abatement emission efforts and pricing decisions. We then make a comparison to judge whose fairness concerns have a greater influence on the supply chain. Finally, we find the conditions under which a revenue-sharing contract can coordinate the supply chain, taking into account members’ fairness concerns under carbon tax regulation.

The remainder of this paper is organized as follows. Section 2 summarizes the relevant literature. Section 3 describes the research problem and assumptions. In Section 4, we investigate the decisions about centralized and decentralized system under carbon tax regulation, taking into account the manufacturer’s and retailer’s fairness concerns. We then compare the manufacturer’s and retailer’s optimal decisions and profits. In Section 5, we propose a revenue-sharing contract to coordinate the supply chain. Section 6 presents numerical examples to illustrate the propositions and reports the analytical results. Conclusions are drawn in Section 7.

## 2. Literature Review

This literature review focuses on three main related areas: (1) research related to carbon emission regulations in supply chain strategic and operational decisions; (2) research related to the effects of fairness concerns on supply chain strategic and operational decisions; and (3) research related to coordination mechanisms on supply chain.

Recently, many papers studied optimization models for supply chain to reduce carbon emissions by affecting the supply chain operations under various carbon emission regulations, such as carbon tax, carbon cap-and-trade, and carbon deposit–refund schemes. Within this realm, there are three different foci. One focus is the logistics management decisions under carbon emission regulations. Konur et al. [18] study an integration inventory control and transportation planning problem by investigating the economic order quantity model with less-than-truckload and truckload transportation under four different carbon emission regulations. Toptal et al. [8] analyze a retailer’s joint decisions on inventory replenishment and carbon emission reduction investment under three carbon emission regulations. He et al. [19] and Ghosh et al. [20] explore issues of production lot-sizing and optimal order quantity under different carbon emission regulations. Hariga et al. [21] present three models to determine the optimal lot sizing and shipping quantities, alongside the number of trucks and freezer units used in the supply chain, which are applied to assess the impacts of carbon tax regulation from transportation and storage activities of a cold item. Song et al. [22] build a two-stage stochastic model to study capacity expansion problem in logistics under cap-and-trade and carbon tax regulations. The second focus is the investigation of impacts of carbon emission regulations on product sustainability design. Dong et al. [9] study the sustainability investment on sustainable product with emission regulation consideration for decentralized and centralized supply chain. Xu et al. [23] compare the sustainability level and sale price of product for decentralize and centralized systems under a cap-and-trade regulation. Xu et al. [24] investigate the production and eco-friendly level of the product decisions of a make-to-order supply chain consisting of a manufacturer and a retailer under cap-and-trade regulation. Yang et al. [7] explore the influence of revenue-sharing and cost-sharing offered by a retailer on manufacturer’s emission abatement effort and their profits, where consumers has environmental awareness and the government levy carbon tax on carbon emission. Besides these, some literatures also study the influences of carbon emission regulations on market competition. Yenipazarli [2] characterizes the optimal emissions taxation policy in order for remanufacturing, and investigates the impact of emissions taxes on the optimal production and pricing decisions of a manufacturer who could remanufacture its own product. Du et al. [25] investigate the behavior and decision making of emission permit supplier and emission-dependent firm by using the newsvendor model. Xu et al. [3] analyze the joint production and pricing problem of a manufacturer with multiple products under cap-and-trade and carbon tax regulations, and compares the effects of the two regulations on the total carbon emissions, the firm’s profits and social welfare. He et al. [26] construct enterprise pricing strategy model and analyze it using the optimization theory and methods under the carbon cap-and-trade regulation with consideration of green technology investment.

Fairness concern is a description of incomplete rationality, which has great impacts on strategic and operational decisions of members in the supply chain. Cui et al. [15] investigate pricing decisions of the supply chain when the manufacturer or retailer is fair-minded, and when both are fair-minded. Du et al. [27] incorporate distribution fairness and carbon emission cap into a supply chain, study the impacts of both factors on supply chain decisions. Yang et al. [28] consider a distribution channel consisting of a single manufacturer and a single retailer, and investigate the effect of the retailer’s fairness concerns on the effectiveness of cooperative advertising. Du et al. [29] explore the effects of relative fairness concern on optimal decisions as well as channel efficiency, and show that the channel efficiency will decrease because of such behavioral preference. Ho et al. [16] and Nie et al. [30] study how distributional and peer-induced fairness might interact and influence economic outcomes in a one-supplier and two-retailer supply chain. Choi et al. [31] examine whether the conclusions of standard supply-chain models (one-shot games) carry over to repeated supply-chain relationships, and find that individual supply chain member’s behavior shows evidence of fairness concerns for supply chain members. Besides the conventional supply chain, some scholars have incorporated fairness concern into the closed-loop supply chain. Ding et al. [32] discuss the pricing decision with retailer’s fairness concern by considering and not considering the retailer’s fairness concern of manufacturers. Liu et al. [33] probe the impacts of remanufacturer’s fairness concern on the optimal product modularity level, production quantity and member’s profits. Ma et al. [17] incorporate retailer’s distributional fairness concerns into the supply chain where the manufacturer is responsible for used-product collection, explore its effects on the optimal marketing effort, collection rate and supply chain performance. Existing literatures have already studied how fairness concerns affect optimal decisions of the supply chain; however, to our best knowledge, there is a scant literature investigating the impacts of fairness concerns on the supply chain under the carbon tax regulation. Our paper studies the decisions of pricing and investment in sustainability under either the manufacturer’s fairness concerns or the retailer’s fairness concerns, and further explores whose fairness concerns have a greater impact on the supply chain.

Supply chain coordination has received considerable attention because some coordination contracts (e.g., whole-price contract, two-part tariffs contract, quantity discount contract, buyback contract, return credit contract, revenue-sharing contract and cost-sharing contract), as demonstrated in [34,35,36,37,38], can improve the performance of the channel. There is limited literature investigating the coordination mechanism under carbon emission regulations. Dong et al. [9] study the coordination of supply chain, and find that only revenue-sharing contract can coordinate the supply chain whereas the buyback contract and two-part tariff contract cannot. Xu et al. [3] consider revenue-sharing and two-tariff contracts to coordinate the sustainable supply chain, and prove the only the two-part tariff can lead to perfect coordination. Yang et al. [7] explore the role of revenue-sharing and first-mover advantage in the manufacturer’s carbon emission abatement effort and the firms’ profitability, their results show that revenue-sharing contract does not necessarily dull the manufacturer’s effort in abatement, it depends on whether the manufacture possesses first-mover advantage and whether consumers have environmental awareness. Xu et al. [24] find that both wholesale price and cost-sharing contracts can coordinate the supply chain, and design a contract in which the retailer pays a lump fee to the manufacture by combining the optimal operational decisions under tow contracts with two-part tariff agreement. Yang et al. [39] explore the efficiency of revenue-sharing and cost-sharing contracts, their results show that both contracts can improve system and manufacturer’s incentive for abatement, and revenue-sharing contract improves over cost-sharing contract, but fails to coordinate them. The aforementioned studies assume that members in the supply chain are completely rational. However, in real life, some players are incompletely rational, and they would be fair-minded for the distribution of profits, especially when they all invest in emission abatement. Therefore, it is meaningful to study coordination mechanisms of the supply chain considering fairness concerns under carbon tax regulation.

## 3. Problem Definition and Assumptions

In this study, we consider a two-echelon sustainable supply chain consisting of one manufacturer and one retailer. The manufacturer is the dominant leader and the retailer is the follower in this two-entity game setting. The manufacturer is responsible for the production of a new product under the government’s carbon tax regulation and pays carbon tax based on the new product’s total carbon emissions. To limit these emissions, the manufacturer increases its investment in the product design stage to improve the sustainability level. The retailer is responsible for selling the new product and conducts low-carbon product promotion through green advertising and other methods.

Under the carbon tax regulation, emissions are treated on a per-unit basis; thus, the government charges the manufacturer a constant carbon tax denoted by $c_{e}>0$. The manufacturer transfers this carbon tax partly to the retailer through the wholesale price, w.

Because of consumers’ low-carbon preference, the manufacturer’s product sustainability level and the retailer’s low-carbon promotion advertising can encourage consumers to purchase products. We denote market demand, D(p,s,h), as D(p,s,h)=φ−p+μs+βh (a similar formulation is proposed in [9,27]), where φ(>0) refers to potential market demand, s(≥0) is the manufacturer’s sustainability level, h(≥0) is the retailer’s low-carbon promotion level, μ(>0) is the coefficient of the sustainability effect on increasing market demand, and β(>0) is the coefficient of the low-carbon promotion effect on increasing market demand. When s=0 (h=0), this indicates that the manufacturer does not invest in product sustainability (the retailer does not invest in the low carbon promotion). The market demand for the product is the complete information for the manufacturer and retailer.

The carbon emissions during production and transportation can be calculated as the carbon emissions per product unit, Q(s)=a−bs, where the sustainability level s is the decision variable, a is the initial carbon emissions, and b measures the reduction of carbon emissions resulting from the manufacturer’s improved sustainability level. A change in product sustainability design is accompanied by an additional fixed cost, $k_{s}$. Following Dong et al. [9], we formulate $k_{s}$ as a quadric form of s, $(\theta s^{2})/2$, where θ(>0) is the sustainability investment coefficient of $k_{s}$ with respect to s, reflecting the ease of investing in product sustainability improvement. In current business practice, where a high level of investment is usually made to improve the product sustainability level, we assume that θ is large enough such that $\theta >\max\{m{\left(\mu +bc_{e}\right)}^{2}/(2m-\beta^{2}),m\mu (\mu +bc_{e})/(m-\beta^{2})\}$. Given the retail price, p, the sustainability level, s, and the low-carbon promotion level, h, the total carbon emissions are J(p,s,h)=Q(s)⋅D(p,s,h).

The retailer conducts low-carbon product promotion through green advertising; moreover, the low-carbon promotion level, h, is the decision variable. The low-carbon promotion cost, $k_{h}$, is the quadric form of h, $(mh^{2})/2$, where m(>0) is the low-carbon promotion advertising coefficient of $k_{h}$ with respect to h, reflecting the ease of investment in green advertising.

In this two-echelon sustainable supply chain, the manufacturer and retailer care not only about their own profits but also about their profits relative to each other. Moreover, this fairness concerning information is common knowledge for the two participating members of the supply chain. In this study, we focus on the two scenarios of fairness concern. (1) The retailer has a fairness concern and the manufacturer is fairness neutral. (2) The retailer is fairness neutral and the manufacturer has a fairness concern. $\lambda_{r}(\geq 0)$($\lambda_{m}(\geq 0)$) denotes the degree of the retailer’s (the manufacturer’s) fairness concern. When $\lambda_{r}(\lambda_{m})=0$, this indicates that the retailer (the manufacturer) has no fairness concern, and when $\lambda_{r}(\lambda_{m})\to \infty$, this indicates that the retailer (the manufacturer) has an extreme fairness concern. In the scenario of the retailer (the manufacturer) with a fairness concern, the retailer (manufacturer) pursues its utility, $u_{R}(u_{M})$, maximization while another participating member manufacturer (retailer) tries to maximize its own individual profit. For the sake of simplicity, we set $\lambda_{r}=\lambda_{m}=\lambda$.

To facilitate the analysis of sustainable supply chain decisions and coordination under carbon tax regulation, taking into account supply chain members’ fairness concerns, we construct a centralized decision model with no fairness concerns, a decentralized decision model with the retailer’s fairness concerns, a decentralized decision model with the manufacturer’s fairness concerns, and a coordination model of a revenue-sharing contract. These are denoted as Models *C*, *R*, *M*, and *FC*, respectively. $\pi_{j}^{i}$ denotes decision-maker j’s profit from Model i and $u_{j}^{i}$ denotes decision-maker j’s utility from Model i, where i={C,R,M,FC} indicates Models *C*, *R*, *M*, and *FC*, and j={R,M,T}. indicates the retailer, manufacturer, and supply chain system, respectively.

In addition, for later convenience, we define the parameters as follows: $\eta =\varphi -ac_{e}-c_{m}$, $A=2m-\beta^{2}$, $B=\mu +bc_{e}$, $C=\mu -bc_{e}$, and $H=A\theta /mB^{2}$, where $c_{m}$ is the unit’s production cost. The symbol η denotes the market demand when the retail price is reduced to the unit’s total cost, namely $ac_{e}+c_{m}$, and, naturally, η>0 is obtained.

## 4. Modeling and Equilibrium Analysis

### 4.1. Model C: The Centralized Decision System

In the centralized decision model, *C*, the manufacturer and retailer operate jointly as a unified firm and the transactions of both parties are the system’s internal behavior. The unified firm determines the optimal retail price, sustainability level, and low-carbon promotion level to maximize the system’s total profit. The total profit function can be formulated as

(1) $$\underset{\left\{p,s,h\right\}}{\max\pi_{}^{C}}=(p-c_{m})D(p,s,h)-c_{e}(a-bs)D(p,s,h)-\frac{1}{2}\theta s^{2}-\frac{1}{2}mh^{2}$$

We can conclude the results with Proposition 1.

**Proposition** **1.** *The unique optimal retail price*, $p^{C*}$, *sustainability level*, $s^{C*}$, *and low-carbon promotion level*, $h^{C*}$, *of the model, C, are as follows:* $p^{C*}=\varphi +\frac{\eta (\theta (\beta^{2}-m)+m\mu B)}{\xi_{c}}$, $s^{C*}=\frac{mB\eta }{\xi_{c}}$, *and* $h^{C*}=\frac{\beta \theta \eta }{\xi_{c}}$, *where* $\xi_{c}=A\theta -mB^{2}$.

For proof, see Appendix A.

In the centralized decision model *C*, the optimal market demand is $D^{C*}=\frac{m\theta \eta }{\xi_{c}}$, the carbon emissions are $J_{}^{C*}=\frac{m\theta \eta }{\xi_{c}}(a-\frac{bm\eta B}{\xi_{c}})$, and the optimal total profit of the centralized system is $\pi_{}^{C*}=\frac{m\theta \eta^{2}}{2\xi_{c}}$.

### 4.2. Model R: The Decentralized Decision System with Retailer’s Fairness Concern

In the scenario of the retailer with a social preference for fairness concern, the retailer pursues a fair profit based on its own strength and contribution, thereby deriving fairness utility. The manufacturer is informed of the retailer’s fairness concern, which the manufacturer should consider when making optimal decisions for profit maximization. The retailer aims to maximize its own fairness utility; thus, its fairness utility function is $u_{R}^{R}=\pi_{R}^{}-\lambda (\pi_{M}^{}-\pi_{R}^{})=(1+\lambda )\pi_{R}^{}-\lambda \pi_{M}^{}$ (a similar formulation is proposed in [28]). The manufacturer’s profit function is $\pi_{M}^{}=(w-c_{m})D(p,s,h)-c_{e}(a-bs)D(p,s,h)-\theta s^{2}/2$ and its decision variables are *wholesale price*, w, and *sustainability level*, s. The retailer’s profit function is $\pi_{R}^{}=(p-w)D(p,s,h)-mh^{2}/2$ and its decision variables are *retail price*, p, and *low-carbon promotion level*, h. Thus, Proposition 2 is as follows.

**Proposition** **2.** *The unique optimal wholesale price*, $w^{R*}$, *retail price*, $p^{R*}$, *sustainability level*, $s^{R*}$, *and low-carbon promotion level*, $h^{R*}$, *of the model, R, are as follows:* $w_{}^{R*}=\varphi +\frac{\eta (m\mu B(1+\lambda )-A\theta (1+3\lambda ))}{\xi_{r}}$, $p_{}^{R*}=\varphi +\frac{\eta (m\mu (1+\lambda )B-\theta (m-\beta^{2})(1+2\lambda ))}{\xi_{r}}$, $s_{}^{R*}=\frac{m\eta B(1+\lambda )}{\xi_{r}}$, *and* $h_{}^{R*}=\frac{\beta \theta \eta (1+2\lambda )}{\xi_{r}}$, *where* $\xi_{r}=2\theta A(1+2\lambda )-mB^{2}(1+\lambda )$.

For proof, see Appendix B.

In the decentralized decision model, *R*, the optimal market demand is $D^{R*}=\frac{m\theta \eta (1+2\lambda )}{\xi_{r}}$ and the total carbon emissions are $J_{}^{R*}=\frac{m\theta \eta (1+2\lambda )}{\xi_{r}}(a-\frac{bm\eta B(1+\lambda )}{\xi_{r}})$. The optimal profits of the manufacturer, retailer, and sustainable supply chain system are $\pi_{M}^{R*}=\frac{m\theta \eta^{2}(1+\lambda )}{\xi_{r}}$, $\pi_{R}^{R*}=\frac{m\theta^{2}\eta^{2}A(1+2\lambda )(1+4\lambda )}{\xi_{r}}$, and $\pi_{T}^{R*}=\frac{m\theta \eta^{2}(3\theta A{\left(1+2\lambda \right)}^{2}-m{\left(1+\lambda \right)}^{2}B^{2})}{2\xi_{r}{}^{2}}$, respectively.

**Corollary** **1.** $s^{R*}$ *and* $h^{R*}$ *decrease with* λ.

For proof, see Appendix C.

When the retailer has a social preference for fairness concern, the manufacturer reduces its wholesale prices and is willing to share some profit with the retailer to limit the supply chain’s loss. The decline of the product’s marginal profit at a lower wholesale price results in the manufacturer investing less in sustainability and thus an inferior product sustainability level. The retailer raises the product’s selling price and reduces the investment in low-carbon promotion in accordance with the principle of utility maximization to achieve a satisfying marginal profit. It can be concluded that the retailer’s fairness concern is not conducive to the sustainable operation of the supply chain.

**Corollary** **2.** *(i) If* 1<H<5/4 *and* λ>2(H−1)/(5−4H), $\pi_{R}^{R*}$ *decreases with* λ *but not* vice versa*; and (ii)* $\pi_{M}^{R*}$ *and* $\pi_{T}^{R*}$ *decrease with* λ.

For proof, see Appendix D.

Corollary 1 shows that the greater the retailer’s fairness concern, the higher the selling price of the product, a situation that will be followed by a decrease in sustainable investment by the supply chain’s participating members. Accordingly, the sales quantity and the manufacturer’s profit dramatically decrease. When 1<H<5/4 and λ>2(H−1)/(5−4H), the product’s marginal profit increases with λ. However, the increment in the marginal profit cannot be offset by the loss incurred by the decline in sales, a situation that will result in the retailer’s profit decreasing. When 1<H<5/4 and λ<2(H−1)/(5−4H) (or H>5/4), the increment in the marginal profit can be offset by the loss incurred by the decline in sales, a situation that will improve the retailer’s profit. Although the increase for the retailer is less than that of the manufacturer, the profit of the supply chain system decreases. Thus, we conclude that the retailer’s fairness concern does not improve the economic benefits of the supply chain.

### 4.3. Model M: The Decentralized Decision System with Manufacturer’s Fairness Concern

As the leader of the sustainable supply chain, the manufacturer takes most of the supply chain’s profit and believes that this allocated proportion is not enough. When the manufacturer has a social preference for fairness concern and aims to maximize its own fairness utility, the fairness utility function can be denoted as $u_{M}^{M}=\pi_{M}^{}-\lambda (\pi_{R}^{}-\pi_{M}^{})=(1+\lambda )\pi_{M}^{}-\lambda \pi_{R}^{}$. The retailer is informed of the manufacturer’s fairness concern, which the retailer should take into account when making optimal decisions for profit maximization. Thus, Proposition 3 is as follows.

**Proposition** **3.** *The unique optimal wholesale price*, $w^{M*}$, *retail price*, $p^{M*}$, *sustainability level*, $s^{M*}$, *and low-carbon promotion level*, $h^{M*}$, *of the model, M, are as follows:* $w_{}^{M*}=\varphi +\frac{\eta (1+\lambda )(m\mu (bc_{e}+\mu )-\theta A)}{\xi_{m}}$, $p_{}^{M*}=\varphi +\frac{\eta (1+\lambda )(\theta (\beta^{2}-m)+m\mu B)}{\xi_{m}}$, $s_{}^{M*}=\frac{m\eta B(1+\lambda )}{\xi_{m}}$, *and* $h_{}^{M*}=\frac{\beta \theta \eta (1+\lambda )}{\xi_{m}}$, *where* $\xi_{m}=\theta A(2+3\lambda )-mB^{2}(1+\lambda )$.

For proof, see Appendix E.

In the decentralized decision model, *M*, the optimal market demand is $D^{M*}=\frac{m\theta \eta (1+\lambda )}{\xi_{m}}$ and the total carbon emissions are $J_{}^{M*}=\frac{m\theta \eta (1+\lambda )}{\xi_{m}}(a-\frac{bm\eta B(1+\lambda )}{\xi_{m}})$. The profits of the manufacturer, retailer, and sustainable supply chain system are $\pi_{M}^{M*}=\frac{m\theta \eta^{2}(1+\lambda )(2A\theta (1+2\lambda )-mB^{2}(1+\lambda ))}{2\xi_{m}{}^{2}}$, $\pi_{R}^{M*}=\frac{mA\eta^{2}\theta^{2}{\left(1+\lambda \right)}^{2}}{2\xi_{m}{}^{2}}$, and $\pi_{T}^{M*}=\frac{m\theta \eta^{2}(1+\lambda )(A\theta (3+5\lambda )-mB^{2}(1+\lambda ))}{2\xi_{m}{}^{2}}$, respectively.

**Corollary** **3.** $s^{M*}$ *and* $h^{M*}$ *decrease with* λ.

For proof, see Appendix F.

To obtain greater marginal profit for the product, the manufacturer raises the wholesale price and reduces investment in product sustainability when its fairness concern is high. The increase in the wholesale price results in the retailer raising the product’s retail price and reducing investment in low-carbon promotion in an attempt to maintain marginal profit. The increasing extent of the effect of the retail price depends on a comparison between θ and m. When m>2θ, the higher low-carbon promotion cost compels the retailer to transfer the total loss of marginal profit to the consumer, namely $\frac{\partial p_{}^{M*}}{\partial \lambda }>\frac{\partial w_{}^{M*}}{\partial \lambda }$. When m<2θ, the retailer transfers part of the loss of marginal profit (which is caused by the manufacturer’s fairness concern) to the consumer, namely $\frac{\partial p_{}^{M*}}{\partial \lambda }<\frac{\partial w_{}^{M*}}{\partial \lambda }$. At the same time, the retailer reduces low-carbon promotion investment to maintain marginal profit. It can be concluded that the manufacturer’s fairness concern is also not conductive to the supply chain’s sustainable operation.

**Corollary** **4.** $\pi_{M}^{M*}$ *and* $\pi_{R}^{M*}$ *decrease with* λ.

For proof, see Appendix H.

Despite the improvement in the product’s marginal profit caused by the manufacturer’s fairness concern, the decline of the retail price, sustainability, and low-carbon promotion investment mainly results in a reduction in the sales quantity; thus, the profits of the manufacturer and retailer decrease. The more the manufacturer exhibits fairness concern, the greater the income loss incurred by the two participating members and the greater the income loss of the supply chain system.

### 4.4. Comparative Analysis of Model Equilibrium

The retailer’s and the manufacturer’s fairness concerns both have significant negative effects on the supply chain’s sustainable operation and economic benefits, although the degree of these effects differs. Propositions 4 and 5 present comparisons of the effects of the fairness concerns of the two players (i.e., the retailer and the manufacturer) on the supply chain sustainability level, low-carbon promotion level, and economic benefits.

**Proposition** **4.** $s^{R*}<s^{M*}$ *and* $h^{R*}>h^{M*}$.

For proof, see Appendix I.

To reduce the adverse effects on the supply chain caused by the retailer’s fairness concern, the manufacturer transfers part of its profit to the retailer by decreasing the wholesale price. Because of the decline in the wholesale price, the manufacturer reduces sustainability investment to maintain marginal profit. Meanwhile, the marginable profit of a new product increases because of the lower wholesale price ($w^{M*}>w^{R*}$) and the higher selling price ($p^{M*}<p^{R*}$). The retailer will not cut the low-carbon promotion investment because a lower level of investment may result in a decrease in market demand. When the manufacturer has a social preference for fairness concern, it gains greater profit from the product with a higher wholesale price and less sustainability level investment. However, the higher wholesale price makes it unnecessary for the manufacturer to reduce sustainability level investment too much because such a reduction merely leads to a slight loss of marginal profit. In addition, $s^{M*}>s^{R*}$ is obtained by taking account of the negative impact of market demand caused by the decline of sustainability investment.

**Proposition** **5.** $\pi_{M}^{M*}<\pi_{M}^{R*}$, $\pi_{R}^{M*}>\pi_{R}^{R*}$, *and* $\pi_{T}^{M*}>\pi_{T}^{R*}$.

For proof, see Appendix G.

When the retailer has a social preference for fairness concern, there are lower levels of sustainability investment; however, the retail price and low-carbon promotion increase. The positive impact of higher low-carbon promotion investment overwhelms the negative impact of the higher selling price and less sustainability investment on market demand, a situation that results in greater market demand for the retailer’s fairness concern than for the manufacturer’s fairness concern. Nonetheless, compared with the scenario involving the manufacturer with a fairness concern, the supply chain enjoys greater retailer profit and total profit, a lower wholesale price, increased sales, and yet a higher level of carbon emissions. Further, the increase in the carbon tax and the decline of the wholesale price reduce the profit of the manufacturer with a fairness concern to a greater extent compared with the scenario involving the retailer with a fairness concern.

## 5. Coordination through a Revenue-Sharing Contract

According to the comparison of the decision variables and the equilibrium solutions of the supply chain between the centralized and decentralized models, we find that the centralized decision model has a higher level of sustainability and low-carbon promotion, greater market demand and profit for the supply chain system, and yet a lower retail price. Each decision-making entity in the decentralized decision model aims to maximize its own profit or utility, which results in double marginalization, non-Pareto optimal profit, and environmental implications. Thus, the manufacturer needs to provide some incentives to encourage the retailer to reduce the selling price and improve low-carbon promotion investment, thereby enabling the profit and environmental implications to achieve the level of the centralized decision model.

To coordinate the two supply chains with the fairness concerns of the manufacturer and retailer, we provide an identical revenue-sharing contract under carbon tax regulation. As an effective and widely used mechanism to coordinate a supply chain, a revenue-sharing contract can be described as follows: The manufacturer sets a lower wholesale price, $w^{FC}$, for the retailer and provides a high level design scheme for sustainability, such that a fraction, Φ(0<Φ<1), of the retailer’s revenue will be transferred to the manufacturer in return and the higher level of low-carbon promotion will be conducted by the retailer.

Under the revenue-sharing contract, the profit functions of the manufacturer and retailer are as follows:

(2) $$\underset{\left\{w,s\right\}}{\max\pi_{M}^{FC}}=(w-c_{m})D(p,s,h)-c_{e}(a-bs)D(p,s,h)-\frac{1}{2}\theta s^{2}+\Phi pD(p,s,h)$$

(3) $$\underset{\left\{p,h\right\}}{\max\pi_{R}^{FC}}=((1-\Phi )p-w)D(p,s,h)-\frac{1}{2}mh^{2}$$

If the revenue-sharing contract achieves the sustainable supply chain’s perfect coordination, the sum of the profits of the manufacturer and retailer will be equal to the supply chain system’s profit derived from the centralized model, namely $\pi_{M}^{FC*}+\pi_{R}^{FC*}=\pi_{T}^{C*}$. Under this condition, $w^{FC*}=c_{m}+ac_{e}$, $s^{FC*}=s^{C*}$, $h^{FC*}=h^{C*}$, and $p^{FC*}=p^{C*}$ hold. Through Equations (2) and (3), we can obtain $\pi_{M}^{FC*}=R_{M}+\Phi S_{R}$ and $\pi_{R}^{FC*}=U_{R}+(1-\Phi )S_{R}$, where $R_{M}=c_{e}bs^{C*}D^{C*}-\theta s^{C*}{}^{2}/2$, $U_{R}=-(c_{m}+ac_{e})D^{C*}-mh^{C*2}/2$, and $S_{R}=p_{}^{C*}D^{C*}$. The symbol $S_{R}$ is the total sales revenue of the retailer under the revenue-sharing contract.

The revenue-sharing contract will be accepted by the decision-maker only when it satisfies the individual rationality constraints so that the sustainable supply chain’s coordination can be realized. When the retailer (manufacturer) has a social preference for fairness concern, the individual rationality constraints are stated as follows: (1) The retailer’s (manufacturer’s) fairness utility from the coordination contract of Model *FC* is no less than the maximum utility of Model *R* (Model *M*), namely $u_{R}^{FC*}\geq u_{R}^{R*}$ ($u_{M}^{FC*}\geq u_{M}^{M*}$). (2) The manufacturer’s (retailer’s) profit from the coordination contract of Model *FC* is no less than that of the optimal Model *R* (Model *M*), namely $\pi_{M}^{FC*}\geq \pi_{M}^{R*}$($\pi_{R}^{FC*}\geq \pi_{R}^{M*}$), where $u_{R}^{FC*}=(1+\lambda )\pi_{R}^{FC*}-\lambda \pi_{M}^{FC*}$($u_{M}^{FC*}=(1+\lambda )\pi_{M}^{FC*}-\lambda \pi_{R}^{FC*}$). According to these inequality groups, we can obtain Proposition 6 as follows.

**Proposition** **6.** *In the presence of the retailer’s fairness concern, when the fraction* Φ *satisfies* $\Phi_{L}^{R}\leq \Phi \leq \Phi_{U}^{R}$*($\Phi_{L}^{M}\leq \Phi \leq \Phi_{U}^{M}$), the two-echelon sustainable supply chain can be coordinated by the revenue*-*sharing contract, where* $\Phi_{L}^{U}=\frac{\left(1+\lambda )(U_{R}+S_{R}-\pi_{R}^{R*})-\lambda (R_{M}-\pi_{M}^{R*}\right)}{(1+2\lambda )S_{R}}$, $\Phi_{L}^{R}=\frac{\pi_{M}^{R*}-R_{M}}{S_{R}}$, $\Phi_{U}^{M}=1-\frac{\pi_{R}^{M*}-U_{R}}{S_{R}}$, *and* $\Phi_{L}^{M}=\frac{\lambda (U_{R}+S_{R}-\pi_{R}^{M*})-(1+\lambda )(R_{M}-\pi_{M}^{M*})}{(1+2\lambda )S_{R}}$.

## 6. Numerical Example

To illustrate our theoretical results more effectively, we use a numerical example in this section to analyze the impact of carbon tax on the equilibrium decisions and profits of supply chain members and to study the efficiency of the revenue-sharing contract. The parameters of the example are set as follows: φ=500, $c_{m}=150$, $c_{e}=12$, μ=0.2, β=0.8, a=8, and b=12.

### 6.1. Analysis of the Impact of Fairness Concern on Equilibrium Decisions and Profits

**Figure 1:**
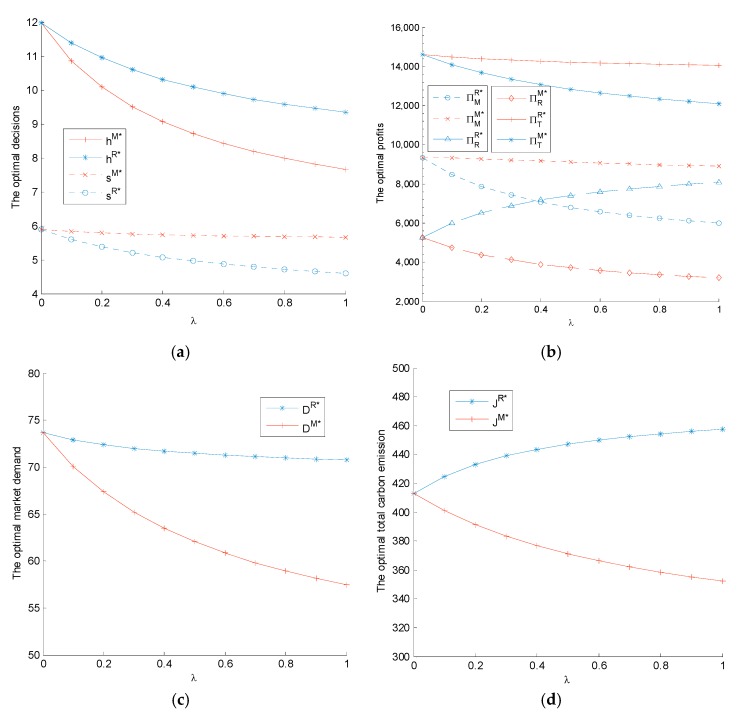
The effects of fairness concern on equilibrium decisions and profits (m=1 and θ=6): (**a**) the optimal sustainability and low-carbon promotion level; (**b**) the optimal profits of the manufacturer, retailer, and supply chain system; (**c**) the optimal market demand; and (**d**) the optimal total carbon emissions.

When the manufacturer’s or retailer’s fairness concern degree λ varies from 0 to 1, the relationship between the equilibrium decisions and profits with fairness concern degree are shown in Figure 1. In Figure 1, we verify that the change trends of optimal decisions, profits, market demand, and total carbon emission are consistent with Corollaries 1–4. Moreover, when player’s fairness concern degree λ=0 i.e. the manufacturer or retailer is fairness neutral, the equilibrium decisions and profits are equivalent to those of the decentralized decision system without player’s fairness concern.

### 6.2. Analysis of the Impact of Carbon Tax on Equilibrium Decisions and Profits

**Figure 2:**
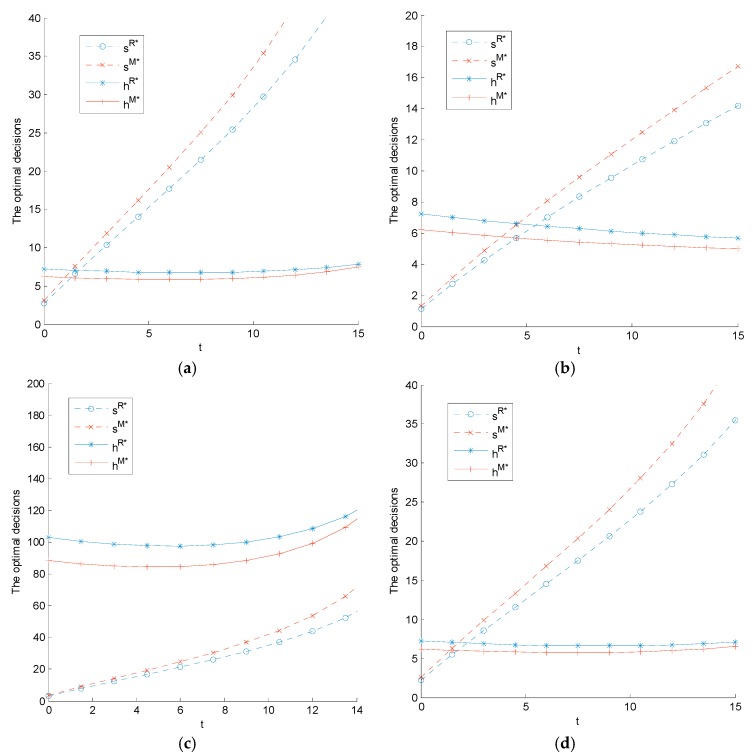
The impact of carbon tax on equilibrium decisions: (**a**) θ=5 and m=10; (**b**) θ=12 and m=10; (**c**) θ=6 and m=1; and (**d**) θ=6 and m=10.

**Figure 3:**
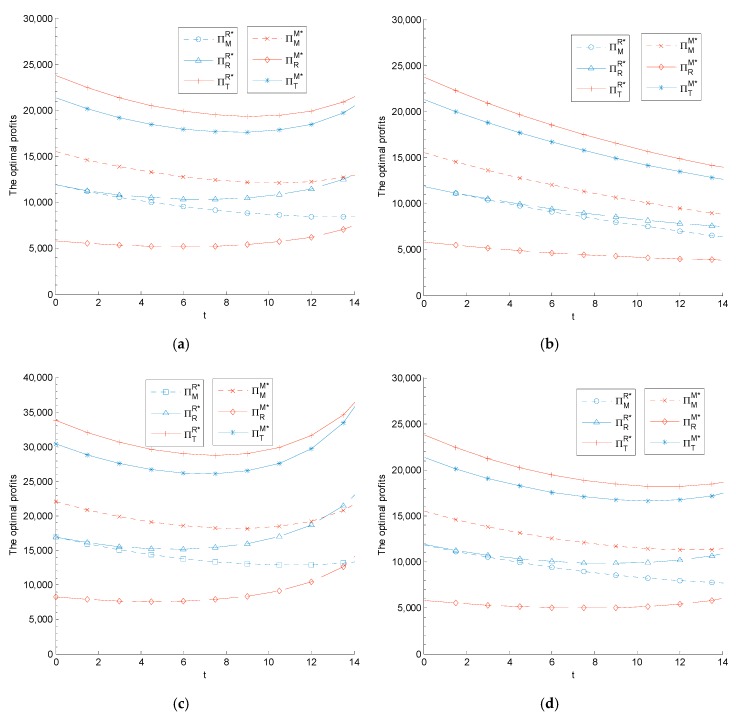
The impact of carbon tax on the supply chain members’ equilibrium profits: (**a**) θ=5 and m=10; (**b**) θ=12 and m=10; (**c**) θ=6 and m=1; and (**d**) θ=6 and m=10.

The impact of carbon tax regulation on the manufacturer’s sustainability level, the retailer’s low-carbon promotion level, and the supply chain members’ profits are related to the parameters m and θ. We set λ=0.5 for different m and θ. The relationship between supply chain members’ sustainable operation levels and profits with carbon tax are shown in Figure 2 and Figure 3.

Figure 2 shows that the manufacturer’s sustainability level is always positively related with carbon tax. Further, its sensitivity to changes in carbon tax is affected by sustainability investment coefficient θ and low-carbon promotion advertising coefficient m. The higher the amounts of θ and m, the smaller the impact of carbon tax on the sustainability level and vice versa. The relationship between the retailer’s low-carbon promotion level and carbon tax largely depends on parameters θ and m. When the manufacturer and retailer have higher sustainable operation costs (when θ and m are both higher), the retailer’s low-carbon promotion level is negatively related with carbon tax; otherwise, the retailer’s low-carbon promotion level is a convex function of carbon tax. There is a carbon tax range, ${\bar{c}}_{e1}=\{{\bar{c}}_{es}^{i},{\bar{c}}_{eh}^{i}\}$, where ${\bar{c}}_{es}^{i}$ and ${\bar{c}}_{eh}^{i}$ denote the carbon tax threshold of the lowest sustainability level and low-carbon promotion level under Model i(i=M,R). When $c_{e}>{\bar{c}}_{es}^{i}({\bar{c}}_{eh}^{i})$, the manufacturer’s sustainability level (the retailer’s low-carbon promotion level) is positively related with carbon tax and vice versa. It can be concluded that the government should identify a threshold when drafting carbon tax regulation to encourage manufacturers (retailers) to increase their product sustainability levels (low-carbon promotion levels). Moreover, the higher the manufacturer’s and retailer’s low-carbon operation costs, the lower the sustainability and low-carbon promotion levels.

The impacts of the two players’ fairness concerns on the supply chain sustainability level differ. Compared with the scenario involving the retailer’s fairness concern, the supply chain enjoys a higher product sustainability level and lower low-carbon promotion level in the scenario with the manufacturer’s fairness concern, a finding that verifies Proposition 4. However, as the carbon tax increases, the value of the low-carbon promotion level is closer to that of the scenario with the retailer’s fairness concern. This finding indicates that the increase in carbon tax can eliminate the negative impact of the manufacturer’s fairness concern on the low-carbon promotion level. Thus, under this condition, the government can set a higher carbon tax than the threshold, ${\bar{c}}_{e}$, a situation that can improve the retailer’s low-carbon promotion level.

Figure 3 shows that the impact of carbon tax on the profits of the manufacturer, retailer, and supply chain system is more relevant to sustainability investment coefficient parameter θ than low-carbon promotion investment coefficient parameter m. Given a higher θ, the profits of the manufacturer, the retailer, and the supply chain system decrease with the increase in the carbon tax. Given a lower θ, there exists a carbon tax threshold, ${\underline{c}}_{e2}=\{{\underline{c}}_{ej}^{i}\}$, under Models *R* and *M*, where ${\underline{c}}_{ej}^{i}$ denotes the corresponding minimum carbon tax threshold of supply chain member j(j=M,R,T) under Model i(i=M,R). When $c_{e}<{\underline{c}}_{ej}^{i}$, the manufacturer’s, retailer’s, and supply chain system’s profits decrease with the increase in the carbon tax and vice versa. Thus, when the carbon tax regulation is being designed, the comparison of ${\bar{c}}_{e1}$ and ${\underline{c}}_{e2}$ should be taken into account by the government because such a comparison plays an important role in motivating the manufacturer (retailer) to raise its product sustainability level (low-carbon promotion level) and promote the supply chain’s economic efficiency.

We find that the influences of decision-makers’ fairness concerns on the profits of each member and the system differ in various scenarios. Compared with the scenario involving the retailer’s fairness concern, in the scenario with the manufacturer’s fairness concern, the manufacturer can obtain a higher profit while the retailer obtains a lower profit, which causes a lower profit for the whole supply chain. These results are consistent with Proposition 5. However, as the carbon tax increases, the system’s profit in the scenario with the manufacturer’s fairness concern will become close to that of the scenario with the retailer’s fairness concern. Thus, the manufacturer’s, retailer’s, and supply chain system’s profits decrease as cost parameters θ and m increase.

### 6.3. Analysis of the Coordination Mechanism’s Efficiency

**Figure 4:**
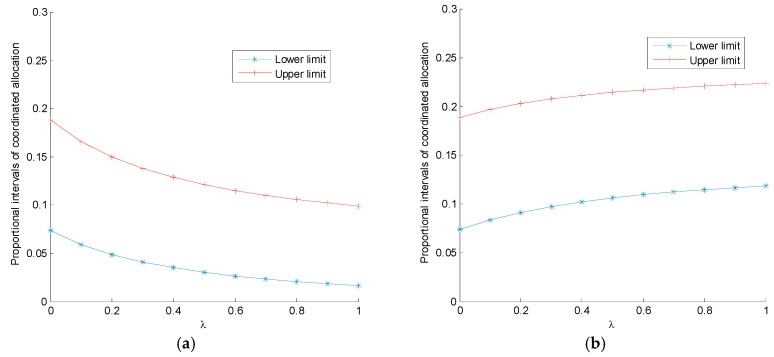

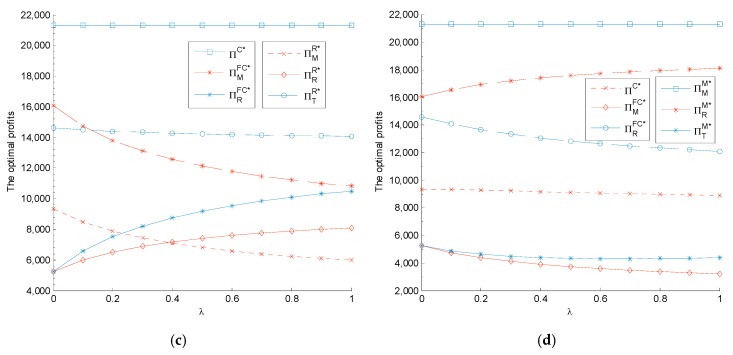
The impact, λ, on the assignment proportion range and the supply chain members’ profits under the revenue-sharing contract: (**a**) coordination assignment range of Model *R*; (**b**) coordination assignment range of Model *M*; (**c**) supply chain members’ profits under the revenue-sharing contract of Model *R*; and (**d**) supply chain members’ profits under the revenue-sharing contract of Model *M*.

Figure 4a shows that both the lower limit, $\Phi_{L}^{R}$, and the upper limit, $\Phi_{U}^{R}$, of the sharing proportion’s effective range, together with $\Phi_{U}^{R}-\Phi_{L}^{R}$, decrease with increasing fairness concern. This finding indicates that as the retailer’s fairness concern increases, it will enjoy greater expected profit; however, the manufacturer will suffer a lower proportion of revenue sharing, which will lead to more difficult coordination. Figure 4b shows that the lower limit, $\Phi_{L}^{M}$, and the upper limit, $\Phi_{U}^{M}$, of the sharing proportion’s effective range increase with rising fairness concern, yet $\Phi_{U}^{M}-\Phi_{L}^{M}$ responds to the contrary. This finding indicates that as the manufacturer’s fairness concern increases, it will expect a higher proportion of the shared revenue, which will lead to more difficult coordination. It can be concluded that the decision-maker’s fairness concern aggravates the supply chain’s double marginalization and increases the difficulty of coordination.

After this analysis, we set $\Phi =\Phi_{U}^{R}(\Phi_{U}^{M})$ and analyze the impact of the revenue-sharing contract on the supply chain members’ profits in the context of the retailer’s (manufacturer’s) fairness concern. Figure 4c,d show that the revenue-sharing contract can coordinate the supply chain perfectly when members have fairness concerns. The retailer provides a higher proportion of the revenue to be shared, the manufacturer has a larger profit increase under the coordination mechanism, and the retailer has a lower profit increase. When the decision-maker is fairness neutral, the coordination mechanism has no effect on the retailer’s profit.

## 7. Conclusions

To control greenhouse gas emissions and slow the pace of global warming, countries worldwide have introduced a series of regulations. These include carbon tax policies whereby the prices of the carbon emissions of enterprises can be fixed. A carbon tax directly affects a firm’s carbon emission costs and sustainability efforts; thus, it affects a product’s price and sales quantity. Consumers’ low-carbon preference and the incomplete rationality of decision-making also affect the firm’s sustainability effort, product pricing, and retail quantity. This study takes into account consumers’ low-carbon preference and analyzes the impacts of the retailer’s (manufacturer’s) fairness concern and carbon tax on the sustainable operation and economic efficiency of a two-echelon supply chain that is coordinated with a revenue-sharing contract. The findings can be summarized as follows.

The retailer’s and manufacturer’s fairness concerns are not conducive to the supply chain’s sustainable operation; moreover, the influences differ. The greater the fairness concerns of the manufacturer and retailer, the lower the manufacturer’s sustainability level and the retailer’s low-carbon promotion level. However, compared with the manufacturer’s fairness concern, the scenario with the retailer’s fairness concern has a lower sustainability level and higher low-carbon promotion level.

The retailer’s and manufacturer’s fairness concerns reduce the supply chain system’s economic efficiency; moreover, the impacts on the members’ profits differ in various scenarios. The manufacturer’s and retailer’s fairness concerns will lead to lower profit for the manufacturer, while the retailer’s profit depends on the different degrees of fairness concern from the various decision-makers. When the manufacturer has a social preference for fairness concern, the retailer’s profit decreases with fairness concern growth. When the retailer has a social preference for fairness concern, of which the value is relatively small, the retailer’s profit increases with fairness concern growth but not vice versa. Moreover, compared with the scenario whereby the manufacturer has fairness concern, the profits of the retailer and the supply chain system are higher, and the profit of the manufacturer is lower.

When the manufacturer or retailer has a social preference for fairness concern, the role that carbon tax regulation plays in terms of the supply chain’s sustainability level and economic efficiency is affected by the investment coefficient parameters of the supply chain’s members. The product sustainability level is always positively correlated with the carbon tax. Moreover, with higher investment coefficient parameters of the manufacturer and retailer, the product sustainability level is more sensitive to carbon tax and vice versa. Only when the investment coefficient parameters of the manufacturer and retailer are both high does the retailer’s low-carbon promotion decrease with the increase in carbon tax; otherwise, there is a non-monotonic convexity function with respect to carbon tax. The impact of carbon tax on the profits of the manufacturer, retailer, and supply chain system relate more to the sustainability investment coefficient parameter than to the low-carbon promotion investment coefficient parameter. When the sustainability investment coefficient parameter is high, then the greater the amount of carbon tax, the lower the profits of the manufacturer, retailer, and supply chain system. When the sustainability investment coefficient parameter is low, the profits of the manufacturer, retailer, and supply chain system are a non-monotonic convexity function of carbon tax. It can be concluded that the government needs to set a suitable carbon tax based on the investment coefficient parameters of supply chain members to promote the supply chain’s economic benefits while at the same time encouraging manufacturers (retailers) to invest in product sustainability levels (low-carbon promotion levels).

Finally, the two-echelon sustainable supply chain is coordinated perfectly by the revenue-sharing contract, which ensures that the optimal sustainability level and economic benefits achieve the level of centralized decision-making. However, the fairness concerns of decision-makers exacerbate the supply chain’s double marginalization and increase the difficulty of coordination.

From the above conclusions, we can infer that fairness concerns of decision makers and the carbon tax significantly influence the operational decisions, economic efficiency and sustainability level of the supply chain. From the policy-maker perspective, the objective of enacting carbon tax is to encourage members to reduce greenhouse gas emissions via enhancing the sustainability of the supply chain. High carbon tax can bring high product sustainability level yet cannot effectively encourage the retailer’s low-carbon promotion. Therefore, the policy-maker needs to set a suitable carbon tax based on the investment coefficient parameters of members in order to promote the product sustainability level (low-carbon promotion level) as well as the economic efficiency of the supply chain. From the manufacturer’s perspective, the fairness concerns of itself is detrimental to its profits, thus, the manufacturer should make optimal decisions for profit maximization rather than fairness utility maximization, and choose the retailer without distributional fairness concerns. From the retailer’s perspective, its fairness concern can strength the bargaining ability and obtain more profits, but the fairness concern is too high to maximize its profits and coordinate the supply chain. Therefore, the retailer should control its level of fairness concern.

In practice, all members included in the supply chain may have fairness concerns. Moreover, carbon regulations such as carbon caps and carbon cap-and-trade are also widely used in real industrial situations. Thus, it would be interesting to investigate the impacts of all members’ fairness concerns on strategic and operational decisions under different carbon emission regulations in the supply chain and, moreover, find more effective and simpler contracts to coordinate the supply chain, taking into account the fairness concerns.

## Appendix A

**Proof of Proposition** **1.** First, we substitute demand function D(p,s,h) into profit function $\pi_{}^{C}$; since $\frac{\partial^{2}\pi^{C}}{\partial p^{2}}=-2$, $\frac{\partial^{2}\pi^{C}}{\partial s^{2}}=2b\mu c_{e}-\theta$, $\frac{\partial^{2}\pi^{C}}{\partial h^{2}}=-m$, $\frac{\partial^{2}\pi^{C}}{\partial p\partial s}=\mu -bc_{e}$, $\frac{\partial^{2}\pi^{C}}{\partial p\partial h}=\beta$, and $\frac{\partial^{2}\pi^{C}}{\partial h\partial s}=b\beta c_{e}$, when $\xi_{c}=\theta A-mB^{2}>0$, the profit function $\pi_{}^{C}$ is strictly joint concave in product price p, sustainability level s and low-carbon promotion level h, therefore the model has the unique optimal solution. Then, through the first-order condition $\frac{\partial \pi_{}^{C}}{\partial p}=0$, $\frac{\partial \pi_{}^{C}}{\partial s}=0$, and $\frac{\partial \pi_{}^{C}}{\partial h}=0$, we have the optimal decisions of Model C as follow, $p^{C*}=\varphi +\frac{\eta (\theta (\beta^{2}-m)+m\mu B)}{\xi_{c}}$, $s^{C*}=\frac{mB\eta }{\xi_{c}}$, and $h^{C*}=\frac{\beta \theta \eta }{\xi_{c}}$.

Proposition 1 is demonstrated.

## Appendix B

**Proof of Proposition** **2.** The game sequence between manufacturer and retailer is a two-stage dynamic game with complete information, and its equilibrium is a sub game perfect Nash equilibrium, so the backward induction method can be used to solve this game problem. First, we substitute demand function D(p,s,h) into retailer’s utility function $u_{R}^{R}$, given the manufacture’s decision variable wholesale price w and sustainability level s. Since $\frac{\partial^{2}u_{R}^{R}}{\partial p^{2}}=-2(1+\lambda )$, $\frac{\partial^{2}u_{R}^{R}}{\partial h^{2}}=-m(1+\lambda )$, and $\frac{\partial^{2}u_{R}^{R}}{\partial p\partial h}=\beta (1+\lambda )$, we have $\frac{\partial^{2}u_{R}^{R}}{\partial p^{2}}\frac{\partial^{2}u_{R}^{R}}{\partial h^{2}}-{\left(\frac{\partial^{2}u_{R}^{R}}{\partial p\partial h}\right)}^{2}=A{\left(1+\lambda \right)}^{2}$. When $A=2m-\beta^{2}>0$, the retailer’s utility function $u_{R}^{R}$ is strictly joint concave in product price p and low-carbon promotion level h, therefore the model has the unique optimal solution. Through the first-order condition $\frac{du_{R}^{R}}{dp}=0$ and $\frac{du_{R}^{R}}{dh}=0$, we can get the retailer’s optimal response functions as $p_{}^{R*}(w,s)=\frac{m(w+s\mu +\varphi )-w\beta^{2}}{A}-\frac{(ac_{e}+c_{m}-bsc_{e}-w)(m-\beta^{2})\lambda }{A(1+\lambda )}$, and $h_{}^{R*}(w,s)=\frac{(ac_{e}+c_{m}-bsc_{e}-w)\beta \lambda }{A(1+\lambda )}+$ $\frac{\beta (\varphi -w+s\mu )}{A}$. We substitute these response functions into the manufacturer’s profit function $\pi_{M}^{R}$. Since $\frac{\partial^{2}\pi_{M}^{R}}{\partial w^{2}}=-\frac{2m(1+2\lambda )}{A(1+\lambda )}$, $\frac{\partial^{2}\pi_{M}^{R}}{\partial s^{2}}=\frac{2mbc_{e}(\mu (1+\lambda )-b\lambda c_{e})}{A(1+\lambda )}-\theta$, and $\frac{\partial^{2}\pi_{M}^{R}}{\partial w\partial s}=\frac{m(\mu (1+\lambda )-bc_{e}(1+3\lambda ))}{A(1+\lambda )}$, we have $\frac{\partial^{2}\pi_{M}^{R}}{\partial w^{2}}\frac{\partial^{2}\pi_{M}^{R}}{\partial s^{2}}-{\left(\frac{\partial^{2}\pi_{M}^{R}}{\partial w\partial s}\right)}^{2}=\frac{\xi_{r}}{A^{2}}$. When $\xi_{r}=2\theta A(1+2\lambda )-mB^{2}(1+\lambda )>0$, the Model R has the unique optimal solutions as follow, $w_{}^{R*}=\varphi +\frac{\eta (m\mu B(1+\lambda )-A\theta (1+3\lambda ))}{\xi_{r}}$, $s_{}^{R*}=\frac{m\eta B(1+\lambda )}{\xi_{r}}$, $h_{}^{R*}=\frac{\beta \theta \eta (1+2\lambda )}{\xi_{r}}$ and $p_{}^{R*}=\varphi +\frac{\eta (m\mu (1+\lambda )B-\theta (m-\beta^{2})(1+2\lambda ))}{\xi_{r}}$. At this point, the optimal product demand and the total manufacture carbon emissions are $D^{R*}=\frac{m\theta \eta (1+2\lambda )}{\xi_{r}}$ and $J_{}^{R*}=\frac{m\theta \eta (1+2\lambda )}{\xi_{r}}(a-\frac{bm\eta B(1+\lambda )}{\xi_{r}})$, respectively.

The optimal benefits of manufacturer, retailer and supply chain system are $\pi_{M}^{R*}=\frac{m\theta \eta^{2}(1+\lambda )}{\xi_{r}}$, $\pi_{R}^{R*}=\frac{m\theta^{2}\eta^{2}A(1+2\lambda )(1+4\lambda )}{\xi_{r}}$, and $\pi_{T}^{R*}=\frac{m\theta \eta^{2}(3\theta A{\left(1+2\lambda \right)}^{2}-m{\left(1+\lambda \right)}^{2}B^{2})}{2\xi_{r}{}^{2}}$, respectively.

Proposition 2 is demonstrated.

## Appendix C

**Proof of Corollary** **1.** Taking partial derivative of optimal solutions $s^{R*}$ and $h^{R*}$ with respect to the fairness concern degree λ, we can get: $\frac{\partial s^{R*}}{\partial \lambda }=-\frac{2m\theta AB\eta }{{\left(\xi_{r}\right)}^{2}}<0$ and $\frac{\partial h^{R*}}{\partial \lambda }=-\frac{m\beta \theta \eta B^{2}}{{\left(\xi_{r}\right)}^{2}}<0$.

Corollary 1 is demonstrated.

## Appendix D

**Proof of Corollary** **2.** Taking partial derivative of optimal profits $\pi_{M}^{R*}$, $\pi_{R}^{R*}$ and $\pi_{T}^{R*}$ with respect to the fairness concern degree λ, we can get: $\frac{\partial \pi_{M}^{R*}}{\partial \lambda }=-\frac{mA\theta^{2}\eta^{2}}{{\left(\xi_{r}\right)}^{2}}<0$, $\frac{\partial \pi_{T}^{R*}}{\partial \lambda }=-\frac{m^{2}A^{2}\theta^{2}B^{2}\eta^{2}(1+4\lambda )}{{\left(\xi_{r}\right)}^{3}}<0$, and $\frac{\partial \pi_{R}^{R*}}{\partial \lambda }=\frac{mA\theta^{2}\eta^{2}(2A\theta (1+2\lambda )-m(2+5\lambda )B^{2})}{{\left(\xi_{r}\right)}^{3}}$. Setting $H=A\theta /mB^{2}$, when 1<H<5/4 and λ>2(H−1)/(5−4H), we get $2A\theta (1+2\lambda )-m(2+5\lambda )B^{2}<0$; that is, $\frac{\partial \pi_{R}^{R*}}{\partial \lambda }<0$. When 1<H<5/4 and λ<2(H−1)/(5−4H) (or H>5/4), we get $2A\theta (1+2\lambda )-m(2+5\lambda )B^{2}>0$, that is $\frac{\partial \pi_{R}^{R*}}{\partial \lambda }>0$.

Corollary 2 is demonstrated.

## Appendix E

**Proof of Proposition** **3.** Similar to the calculation of the decision Model R, according to the backward induction method, when $A=2m-\beta^{2}>0$, the retailer’s optimal response functions are $p_{}^{M*}(w,s)=\frac{m(w+s\mu +\varphi )-w\beta^{2}}{A}$ and $h_{}^{M*}(w,s)=\frac{\beta (\varphi -w+s\mu )}{A}$. Substituting these response functions into the manufacturer’s utility function $u_{M}^{M}$, since $\frac{\partial^{2}u_{M}^{M}}{\partial w^{2}}=-\frac{m(2+3\lambda )}{A}$, $\frac{\partial^{2}u_{M}^{M}}{\partial s^{2}}=\frac{m\mu (2bc_{e}(1+\lambda )+\lambda \mu )}{A}-\theta (1+\lambda )$, and $\frac{\partial^{2}u_{M}^{M}}{\partial w\partial s}=\frac{m(\mu (1+2\lambda )-bc_{e}(1+\lambda ))}{A}$, we can get $\frac{\partial^{2}u_{M}^{M}}{\partial w^{2}}\frac{\partial^{2}u_{M}^{M}}{\partial s^{2}}-{\left(\frac{\partial^{2}u_{M}^{M}}{\partial w\partial s}\right)}^{2}=\frac{m\xi_{m}}{A^{2}(1+\lambda )}$. When $\xi_{m}=\theta A(2+3\lambda )-mB^{2}(1+\lambda )>0$, the Model M has the unique optimal solutions as follow, $w_{}^{M*}=\varphi +\frac{\eta (1+\lambda )(m\mu (bc_{e}+\mu )-\theta A)}{\xi_{m}}$, $s_{}^{M*}=\frac{m\eta B(1+\lambda )}{\xi_{m}}$, $p_{}^{M*}=\varphi +\frac{\eta (1+\lambda )(\theta (\beta^{2}-m)+m\mu B)}{\xi_{m}}$, and $h_{}^{M*}=\frac{\beta \theta \eta (1+\lambda )}{\xi_{m}}$. At this point, the optimal product demand and the total manufacture carbon emissions are $D^{M*}=\frac{m\theta \eta (1+\lambda )}{\xi_{m}}$ and $J_{}^{M*}=\frac{m\theta \eta (1+\lambda )}{\xi_{m}}(a-\frac{bm\eta B(1+\lambda )}{\xi_{m}})$, respectively.

The optimal benefits of retailer, manufacturer and supply chain system are $\pi_{R}^{M*}=\frac{mA\eta^{2}\theta^{2}{\left(1+\lambda \right)}^{2}}{2\xi_{m}{}^{2}}$, $\pi_{M}^{M*}=\frac{m\theta \eta^{2}(1+\lambda )(2A\theta (1+2\lambda )-mB^{2}(1+\lambda ))}{2\xi_{m}{}^{2}}$, and $\pi_{T}^{M*}=\frac{m\theta \eta^{2}(1+\lambda )(A\theta (3+5\lambda )-mB^{2}(1+\lambda ))}{2\xi_{m}{}^{2}}$, respectively.

Proposition 3 is demonstrated.

## Appendix F

**Proof of Corollary** **3.** Taking partial derivative of optimal solutions $s^{M*}$ and $h^{M*}$ with respect to the fairness concern degree λ, we can get: $\frac{\partial s^{M*}}{\partial \lambda }=-\frac{m\theta AB\eta }{{\left(\xi_{m}\right)}^{2}}<0$ and $\frac{\partial h^{M*}}{\partial \lambda }=-\frac{mA\eta \theta^{2}}{{\left(\xi_{r}\right)}^{2}}<0$.

Corollary 3 is demonstrated.

## Appendix G

**Proof of Corollary** **4.** Taking partial derivative of optimal profits $\pi_{M}^{M*}$ and $\pi_{R}^{M*}$ with respect to the fairness concern degree λ, we can get: $\frac{\partial \pi_{M}^{M*}}{\partial \lambda }=-\frac{m\lambda A^{2}\theta^{3}\eta^{2}}{{\left(\xi_{m}\right)}^{2}}<0$ and $\frac{\partial \pi_{T}^{R*}}{\partial \lambda }=-\frac{mA^{2}\theta^{3}\eta^{2}(1+\lambda )}{{\left(\xi_{r}\right)}^{3}}<0$.

Corollary 4 is demonstrated.

## Appendix H

**Proof of Proposition** **4.** According to the expression of the equilibrium solution, subtract the optimal decision corresponding to the Model M and Model R, we can infer that $s_{}^{M*}-s_{}^{R*}=\frac{m\theta \lambda \eta AB(1+\lambda )}{\xi_{r}\xi_{m}}>0$, $p_{}^{M*}-p_{}^{R*}=$ $\frac{\theta \lambda \eta (((m-\beta^{2})b^{2}c_{e}{}^{2}-\mu (bc_{e}\beta^{2}+m))m(1+\lambda )-(m-\beta^{2})A\theta (1+2\lambda ))}{\xi_{r}\xi_{m}}>0$, $h_{}^{R*}-h_{}^{M*}=\frac{2\beta \theta \eta (\theta A(1+2\lambda )-mB^{2}(1+\lambda ))}{\xi_{r}\xi_{m}}>0$, and $D_{}^{R*}-D_{}^{M*}=\frac{m\theta \eta (\theta A(1+2\lambda )-mB^{2}(1+\lambda ))}{\xi_{r}\xi_{m}}>0$.

Proposition 4 is demonstrated.

## Appendix I

**Proof of Proposition** **5.** According to the expression of the equilibrium solution, subtract the optimal profit corresponding to the Model M and Model R, we can infer that $\pi_{M}^{M*}-\pi_{M}^{R*}=\frac{m\lambda \theta^{2}\eta^{2}(1+\lambda )(\theta A(4+7\lambda )-2mB^{2}(1+\lambda ))}{2\xi_{r}\xi_{m}^{2}}>0$, $\pi_{R}^{R*}-\pi_{R}^{M*}=\frac{mA\theta^{2}\eta^{2}((1+2\lambda )(1+4\lambda )\xi_{m}^{2}-{\left(1+\lambda \right)}^{2}\xi_{r}^{2})}{2\xi_{r}^{2}\xi_{m}^{2}}>0$, and $\pi_{T}^{R*}-\pi_{T}^{M*}=$ $\frac{m\theta \eta^{2}(\xi_{m}^{2}(3\theta A{\left(1+2\lambda \right)}^{2}-mB^{2}{\left(1+\lambda \right)}^{2})-\xi_{r}^{2}((1+\lambda )(\theta A(3+5\lambda )-mB^{2}(1+\lambda ))))}{2\xi_{r}^{2}\xi_{m}^{2}}>0$.

Proposition 5 is demonstrated.
